# Primary Peritoneal Serous Cancer: A Case Report of a Tumor in the Rectovaginal Septum

**DOI:** 10.1155/2024/5093727

**Published:** 2024-01-16

**Authors:** Analy Herrera-Torres, Carlos Guadalupe Parra-Torres, Gabriela C. Alamilla García, María del Rocío Thompson Bonilla, Oscar Manuel García Córdova, Alfredo Padilla Martínez, Rodolfo Iván Lara Ruíz, Esther Ramírez Moreno

**Affiliations:** ^1^Institute for Social Security and Services for State Workers, Hospital Regional 1° de Octubre, Mexico City, Mexico; ^2^Instituto Politécnico Nacional, Escuela Nacional de Ciencias Biológicas USEIC, Mexico City, Mexico; ^3^Instituto Politécnico Nacional, Escuela Nacional de Medicina y Homeopatía, Mexico City, Mexico

## Abstract

Peritoneal cancer is the invasion by malignant cells of serous membrane that lines the abdominal cavity, the viscera, and the coelom of the amniotes. Histologically, it is indistinguishable from ovarian counterpart, although in the former, it commonly involves the ovary only superficially, or it may totally lack an ovarian component, but with extensive involvement of the peritoneum, calcified perihepatic peritoneal nodules, or involvement of the omentum, in most cases. The current study describes the case of a 54-year-old female patient referring a history of colitis and dairy intolerance. A transvaginal ultrasound and a computed tomography (CT) scan revealed a tumor measuring 70 × 61 × 63 mm. CA-125 serum levels were 880 U/ml. Laparotomy surgery was indicated, and tumor was found at the level of the rectovaginal septum without evidence of metastasis. Tumor dissection and protective colostomy with loop sigmoid colon were performed. A pathological study gave a diagnosis of a high-grade peritoneal serous carcinoma with a micropapillary pattern. The present study describes the case of papillary serous peritoneal cancer presented as a single tumor mass without extensive involvement of the peritoneum. Additionally, the need for routine tests for its diagnosis and documenting hormonal alterations as the cause of its origin are suggested.

## 1. Introduction

Peritoneal cancer or malignancy of the peritoneal surface is the invasion by malignant cells, of serous membrane that lines the abdominal cavity, the viscera, and the coelom in amniotes; it is divided in primary and secondary types. The *de novo* origin of cancer in the abdominal mesothelium produced primary mesothelioma, and on the contrary, dissemination of tumor cells into the peritoneal cavity from other sites results in secondary peritoneal cancer.

Physicians classified the primary cancer based on its histology, and extraovarian primary peritoneal carcinoma (EOPPC), serous surface papillary carcinoma, serous papillary carcinoma of the peritoneum, extraovarian Mullerian adenocarcinoma, and normal-size ovarian carcinoma syndrome are different terms used for the primary peritoneal cancer. This carcinoma is rare and occurs principally in older postmenopausal women [1]. In addition, the most common histological type of primary peritoneal cancer is serous carcinoma of the peritoneum and represents 10% of cancers occurring in the pelvis [2].

In 1954, Swerdlow published a clinical case evidencing the primary origin of a tumor from the pelvic peritoneum, which he called “pelvic peritoneal mesothelioma.” This occurred in a 27-year-old woman with an abdominal tumor, histologically similar to an ovarian papillary cystadenocarcinoma; however, ovaries did not show alterations, and careful examination showed no other tumor masses in the abdominal cavity or in any of the viscera within it. The author suggested that some inflammatory or hormonal influence could induce the pelvic peritoneum, “genitally mature” and with its stored “embryonic” potential, to differentiate or resume its embryonic function producing an endometrial cyst or dedifferentiating and producing a papillary tumor [3].

The mesothelium of the peritoneum and the germinal epithelium of the ovary have the same embryonic origin, so the peritoneum can retain the multipotentiality of the Mullerian system and allow the emergence of a primary carcinoma that is similar in histological appearance, dissemination, treatment, and prognosis to the papillary serous ovarian carcinoma [4]. Histologically, primary papillary carcinoma of peritoneum is indistinguishable from the ovarian counterpart, although in the former, it commonly involves the ovary only superficially, or it may totally lack an ovarian component [5].

### 1.1. Risk Factors

#### 1.1.1. General Factors

There are different factors associated with the risk of having peritoneum cancer or cancers with a similar origin, among them are having direct relatives who have suffered from this disease, taking hormone replacement therapy after menopause, being overweight or obese, and having endometriosis [6].

Inflammation is also considered an important risk factor for high-grade epithelial and serous ovarian cancer. Overweight, obesity, endometriosis, and other factors that produce a systemic or local inflammatory condition have also been associated with an increased risk of ovarian cancer. Chronic inflammation results in the activation of signaling pathways, transcription factors, and dysregulation of cytokine secretion that, in turn, potentiates the initiation of normal cells to malignant ones and supports tumor progression, metastasis, and development of resistance to chemotherapy [7, 8].

#### 1.1.2. Genetic Factors

A genetic risk factor associated to peritoneal carcinoma is the presence of mutations in the BRCA- (breast cancer-) 1 and BRCA-2 genes in the germline DNA [9–11]; however, the date is still not totally conclusive. On the other hand, a molecular alteration associated with peritoneal carcinoma is the presence of mutations in the p53 gene, which are identical in all disseminated tumors of patients with serous peritoneal carcinoma, supporting the hypothesis about the monoclonal origin of dissemination [12]. Likewise, it has been documented that there is a double expression of the HER-2/neu (human epidermal growth factor receptor 2) oncogene in peritoneal cancer compared to ovarian cancer [13].

#### 1.1.3. Hormonal Factors

Sex steroids have been widely described to be associated with a number of human diseases, including hormone-dependent tumors. In premenopausal women, large fluctuations in the concentration of circulating estradiol (E2) and progesterone (P4) orchestrate many events across the menstrual cycle. After menopause, the levels of circulating E2 and P4 decline but remain at a high concentration in the peripheral tissues. Notably, there is a strong relationship between circulating sex hormones and female reproductive cancers (e.g., ovarian, breast, and endometrial cancers). These hormones activate a number of specific signaling pathways after binding either to estrogen receptors (ERs), especially ER*α*, ER*α*36, and ER*β* or progesterone receptors (PRs), which response may be associated with the risk of malignancy ([14].

Another hormone related to oncogenic processes is prolactin, a hormone produced by the pituitary gland and multiple extrapituitary sites, since it is known that it and its receptor activate a series of effects such as survival, cell proliferation, migration, invasion, metastasis, and resistance to treatment. Several findings point out the relevance of prolactin in ovarian cancer development. Prolactin could induce carcinogenesis by regulating gene expression, for instance, CD24+, whose overexpression is associated with the tumor development and metastasis of human cancers [15, 16]. Other observations hypothesize that prolactin activates PI3K/Akt and induces the expression of antiapoptotic genes, such as Bcl-2, which is seen in different types of cancers. Also, extrapituitary prolactin, which acts mainly in an autocrine and paracrine manner, has been an essential survival factor for ovarian cancer by inhibiting apoptosis in these cells [17].

### 1.2. Epidemiology

In Mexico, the national epidemiological information regarding the incidence of the different types of cancer is very limited; therefore, there are no epidemiological data on peritoneum cancer, and existing information is focusing on ovarian cancer. For example, recently, the incidence of cancer in the state of Mérida was reported between the years 2015 to 2018, registering a total of 5,684 cases, with 836 cases of genital cancer in women (14.7%), with an incidence rate standardized per age of 8.34 per 100,000 inhabitants for ovarian and adnexal cancer [18]. Additionally, using projections of the national population in Mexico and available statistical data on cancer, it was found that the mortality rate of women due to ovary cancer increased from 2.26 to 3.41/100,000 women between the years 2000 and 2013, which suggests an increase in incidence [19].

In the United States, it was reported that between the years 1995 and 2004, the incidence of peritoneal carcinoma was 6.78 cases per million individuals, being minor to the incidence of ovarian cancer (119.9 cases); white women had the highest rates of these malignancies, while peritoneal carcinoma had the lowest rate among dark-skinned women (2.88 cases per million). Serous carcinoma was the most frequently diagnosed histological type in the ovary, peritoneum, and fallopian tube. Analysis of trends between 1973 and 2005 exhibited a significant decrease in the incidence of ovarian carcinoma and an increase in the rate of peritoneal cancer [20].

### 1.3. Screening

Peritoneal cancer is usually diagnosed in late stages, due symptomatology is not very specific or evident, and is often confused with gastrointestinal disorders. Symptoms related with all peritoneal carcinomas are abdominal swelling, bloating, nausea, indigestion, anorexia, weight loss, fatigue, constipation, and abdominal or back pain. Most cases are misdiagnosed as epithelial ovarian cancer. Peritoneal carcinomatosis produced symptom characteristic of the primary tumor and nonspecific symptoms. Secondary metastatic deposits can range from microscopic involvement to nodules and bulky disease; the symptoms are determined by the degree of involvement and its location. The growth of primary and secondary tumors causes pressure effects that result in mechanical intestinal obstruction [21].

Screening test for ovarian, fallopian tube, and primary peritoneal cancer includes pelvic exam, transvaginal ultrasound and CA-125 antigen evaluation. However, although CA-125 is elevated in 82% of women with advanced ovarian cancer, it has very limited clinical application for the detection of early-stage disease. New strategies are being developed for the early detection of ovarian cancer (which is useful for cancers that form in the same type of tissue, such as peritoneum cancer), based on the analysis of serum biomarkers whose abundance changes during illness. Combinations of these biomarkers, such as leptin, prolactin, osteopontin, and insulin-like growth factor [22], or MSP-alpha, TIMP-4, PDGF-R alpha, and OPG and CA-125 [23], have been tested, increasing the specificity and sensitivity of these early detection tools.

### 1.4. Treatment

A combination of surgery, chemotherapy, and targeted therapy is the mainstay of treatment for peritoneal cancer. Chemotherapy includes systemic and peritoneal chemotherapy with poly (ADP-ribose) polymerase (PARP) inhibitors that block DNA repair. It includes olaparib, rucaparib, niraparib, or veliparib [21]. Extensive debulking is required in 98% of cases for abdominal wall tumor and pelvic disease. Surgeries most include a hysterectomy with bilateral salpingoophorectomy and omentectomy. Chemotherapeutic agents for primary peritoneal cancer have been found to be those that have been effective for advanced epithelial ovarian cancer. Current recommendations include the combination of taxanes with carboplatin (paclitaxel and carboplatin) [24].

## 2. Case Report

**Figure 1:**
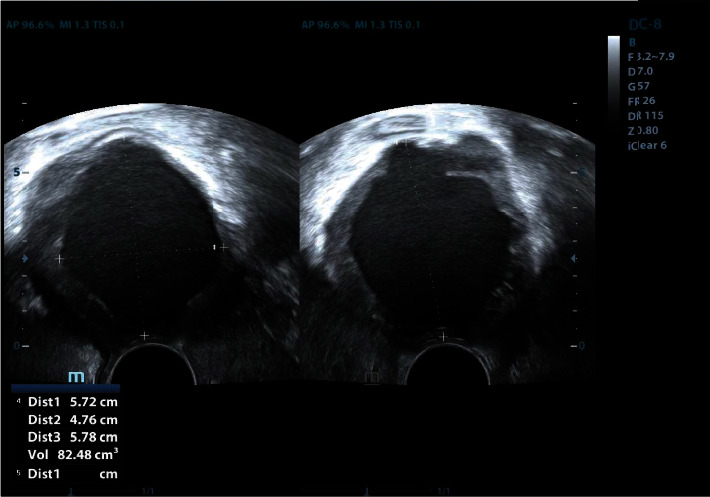
Transvaginal ultrasound image showing a tumor with dimensions of 57.2 × 47.6 × 57.8 mm.

**Figure 2:**
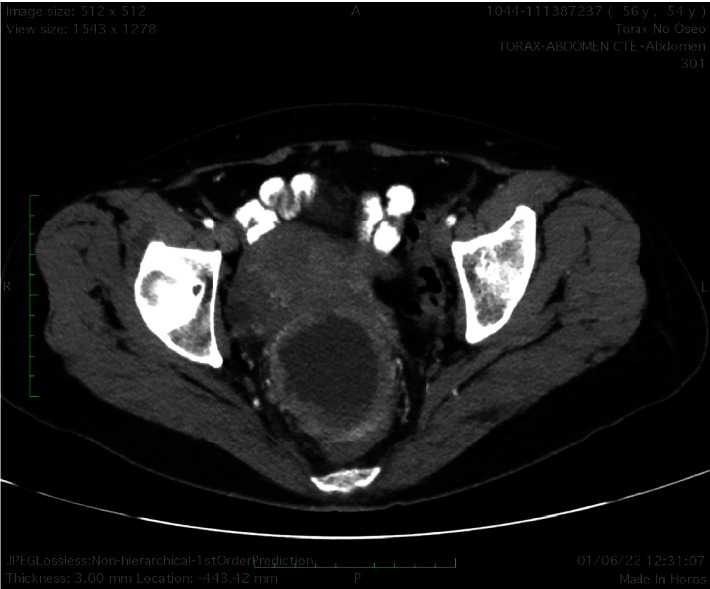
Contrast-enhanced computed tomography (CT) scan revealing a cystic tumor. Axial slice of a contrasted tomography of the abdomen visualized at the level of the pelvis where a circumscribed hypodense image is observed, and with a thick wall with enhancement to the contrast, as well as loss of the interface with the uterus and rectum, both are displaced.

The patient is a 54-year-old Mexican woman, who has a history of colitis and dairy intolerance but was in good general physical condition; she attends her annual gynecological checkup, after two years of not attending due to the COVID-19 pandemic. A cyst in the right ovary with benign characteristics was identified by abdominal ultrasound, after which CA-125 antigen evaluation was indicated as a precautionary measure, along with routine studies. CA-125 was found with a value of 880 U/ml (reference 0-35 U/ml), so a transvaginal ultrasound was performed, identifying a tumor measuring 57.2 × 47.6 × 57.8 mm with a heterogeneous echotexture, exhibiting irregular vegetating internal margins and containing fine mobile echogenic elements, considering characteristics suspicious for malignancy (Figure 1). The patient was referred to the surgical oncology service of the 1^o^ de Octubre Hospital-ISSSTE, Mexico, where a contrast-enhanced computerized tomography (CT) scan confirmed the presence of a tumor with measures of 70 × 61 × 63 mm, located in the pelvis (Figure 2). Laparotomy surgery was indicated. The cavity was checked without evidence of metastasis, a 4 cm cyst was found in the right ovary and the uterus, displaced due to tumoral shift, was of normal size (7 × 4 × 3 cm) with a mildly distended endometrial cavity; hysterectomy and salpingoophorectomy were performed without complications. Adhesions and a tumor were found at the level of the rectovaginal septum. The tumor was dissected, including the compromised region of the vagina and part of the external layer of the sigmoid rectum, for which a protective colostomy with a sigmoid colon loop was performed.

Pathological study gave a diagnosis of high-grade peritoneal serous carcinoma with micropapillary pattern infiltrated of the entire vaginal wall and lymph vascular permeation, lesion size 5 × 4 cm in contact with the Douglas pouch, peritoneal fluid positive to neoplastic cells, vaginal border without lesion, cervix without atrophy, parametria without evidence of neoplasia, basal endometrium, ovaries without white bodies, and salpinx without alterations.

**Figure 3:**
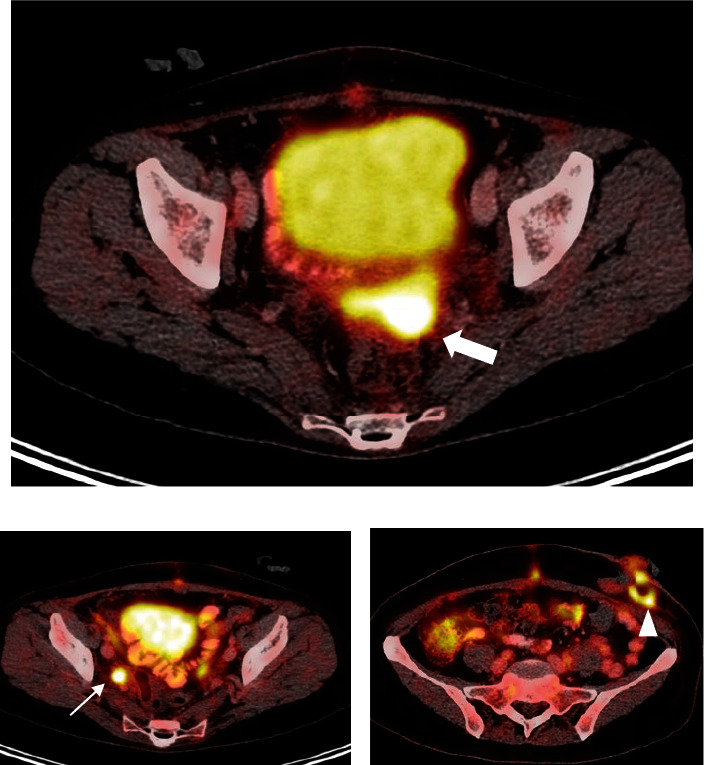
Positron emission tomography/computed tomography (PET-CT) July 2022. The white arrow indicates hot uptake in the vaginal dome (a), the thin arrow shows right adenopathy (b), and the arrowhead shows hypermetabolism at the stoma site compatible with an infectious process (c).

One month after surgery, a positron emission tomography/computed tomography (PET-CT) scan was performed (July 2022) to detect areas with increased metabolic activity, related with the presence of residual tumor tissue. It detected hypermetabolic tumor activity at the level of the vaginal dome, right obturator adenopathy, referred peritoneal implant, and left focalized functional colostomy with incipient infectious process (Figure 3).

Two months after recovery, the patient received chemotherapy treatment consisting of six cycles of paclitaxel (240 mg) and carboplatin (600 mg), every 21 days. At the beginning of the treatment, the CA-125 value was 388 U/ml and decreased to 7.8 U/ml at the end of the treatment; it is worth mentioning that the CA-125 values decreased to normal values since the patient received the second cycle of chemotherapy.

**Figure 4:**
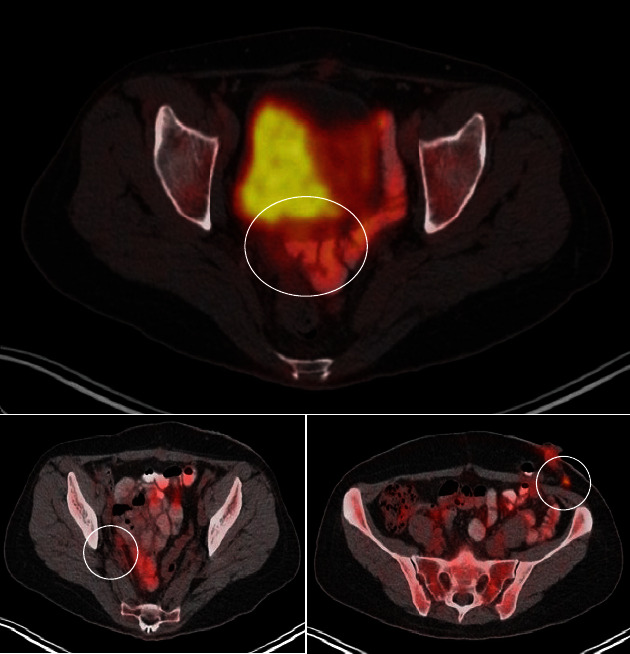
Positron emission tomography/computed tomography (PET-CT) findings postchemotherapy (February 2023). Circles show areas without hypermetabolism, compared to the previous PET-CT analysis.

A second PET-CT was performed 23 days after the last chemotherapy cycle (February 2023); it was found that there was no evidence of hypermetabolic tumor activity, compared to the previous study, and data were in relation to complete response (Figure 4).

Laparotomy was scheduled two months later, for a second look, and intestinal reconnection. No alterations were observed in organs, such as the peritoneum, bowels, diaphragm, bladder, or vagina, which corresponds to what was observed in the PET-CT. It was decided to follow up the patient by measuring CA-125 levels every two months and by PET/CT at six months. At the time of this publication, the patient has a survival of 18 months after surgery (June 2022-December 2023).

## 3. Discussion

Due to primary peritoneal cancer being a rare condition and whose diagnosis is made late, it is necessary to implement strategies that allow its timely detection. In Mexico, the public and private health system contemplates routine tests for the early diagnosis of the most frequent cancers in the female population, such as breast and cervical cancer. These include annual radiographic studies of the breast and Papanicolaou tests and colposcopy; however, they do not consider the detection of alterations in the peritoneum, ovaries, or the abdominal cavity, for which it is a priority to have a periodic detection scheme that includes an abdominal or transvaginal ultrasound, and the measurement of CA-125 antigen in blood samples. These tests, which are neither invasive nor expensive, would allow the detection of primary peritoneal cancer and other cancers in the abdominal cavity, avoiding the inherent complications of a late detection that impact in the quality and life expectancy of patients, and avoiding economic expenses for the treatment and care of cancer patients in public and private hospitals.

Cases of primary peritoneal cancer are very rare and are often misdiagnosed as epithelial ovarian cancer. The clinical case of primary peritoneal cancer that we present here consists of a single tumor located at the level of the rectovaginal septum, but without extensive involvement of the peritoneum, without calcified perihepatic peritoneal nodules, or involvement of the omentum, as what occurs in most cases. A similar case was described for the first time in 1959 in a 27-year-old patient, with a tumor located in the left adnexal region of the abdomen. The author suggests that the tumor could have developed due to a hormonal or inflammatory influence, activating the embryonic potential of peritoneal cells [3].

In this regard, the current patient mentions having had hyperprolactinemia with galactorrhea at 34 years of age, which was controlled when she managed to get pregnant. Unfortunately, her prolactin levels were not monitored afterwards, and it is not known if this hormonal imbalance was temporary or chronic. Interestingly, there are scientific evidences supporting the hypothesis that the local accumulation of prolactin may contribute to the tumorigenesis of breast, colorectal [25], and high-grade serous ovarian cancer [26]. Also, the activation of prolactin receptor is known to activate several kinases that stimulate cell proliferation [27].

Since there are no well-defined risk factors in primary peritoneal cancer, it will be very useful to find the relationship of this and other tumors with hormonal changes such as hyperprolactinemia and/or inflammatory processes.

Finally, it is important to highlight that the response to chemotherapy with carboplatin and paclitaxel in this clinical case was very favorable, with no evidence of malignancy remaining at the end of treatment, which was determined by PET-CT and laparotomy. The follow-up of this patient is aimed at contributing to the recording of the survival time of this pathology, since these data are very scarce in the clinical cases published in the literature.

In conclusion, since primary peritoneal cancer is a very rare malignancy and difficult to detect, it is convenient to implement easily accessible routine tests that allow its early detection in the female population. Likewise, it is a priority to determine if hormonal factors could be associated with its origin, which would lead to finding preventive therapies and therapeutic alternatives.
